# Troubleshooting Guide to Expressing Intrinsically Disordered Proteins for Use in NMR Experiments

**DOI:** 10.3389/fmolb.2018.00118

**Published:** 2019-01-18

**Authors:** Steffen P. Graether

**Affiliations:** Department of Molecular and Cellular Biology, University of Guelph, Guelph, ON, Canada

**Keywords:** intrinsically disordered proteins (IDPs), NMR, expression, isotopic labeling, purification, optimization, structure

## Abstract

Intrinsically disordered proteins (IDPs) represent a structural class of proteins that do not have a well-defined, 3D fold in solution, and often have little secondary structure. To characterize their function and molecular mechanism, it is helpful to examine their structure using nuclear magnetic resonance (NMR), which can report on properties, such as residual structure (at both the secondary and tertiary levels), ligand binding affinity, and the effect of ligand binding on IDP structure, all on a per residue basis. This brief review reports on the common problems and decisions that are involved when preparing a disordered protein for NMR studies. The paper covers gene design, expression host choice, protein purification, and the initial NMR experiments that are performed. While many of these steps are essentially identical to those for ordered proteins, a few key differences are highlighted, including the extreme sensitivity of IDPs to proteolytic cleavage, the ability to use denaturing conditions without having to refold the protein, the optimal chromatographic system choice, and the challenges of quantifying an IDP. After successful purification, characterization by NMR can be done using the standard ^15^N-heteronuclear single quantum coherence (^15^N-HSQC) experiment, or the newer CON series of experiments that are superior for disordered proteins.

## Introduction

Intrinsically disordered proteins (IDPs, also known as intrinsically unstructured proteins or natively unfolded proteins) are a relatively recently identified class of structures with many properties that often go against the dogma of structural biology (Wright and Dyson, 1999; Uversky et al., 2000; Dunker et al., 2001; Tompa, 2002; Uversky, 2002a). Alone in solution, IDPs have no fixed 3D fold, but instead are better described as “boiling spaghetti” (Uversky, 2013) or “protein clouds” (Uversky, 2016). Despite their lack of structure, disordered proteins have specific functions, and are able to bind ligands with specificity yet at a low affinity (Uversky et al., 2008). Some IDPs gain structure in the presence of their ligand, sometimes even having different structures in the presence of different ligands (Fuxreiter and Tompa, 2012).

There is great research value in determining the “structure” of an IDP despite its disorder; firstly and simply, analysis of a putative IDP will experimentally confirm that it is in fact disordered, or even suggest what fraction and/or regions of the protein are disordered. Secondly, it is estimated that ~20% of proteins encoded in higher eukaryotic genomes are disordered (Oldfield et al., 2005), and yet the structures of only a small number of IDPs have been studied in detail (Varadi et al., 2014). Clearly, there is considerably more information we need to learn before we can understand how these fascinating proteins function.

This brief troubleshooting guide outlines the problems that may be encountered during expression and purification of IDPs that will be characterized by nuclear magnetic resonance (NMR) experiments; the flexibility of IDPs makes it essentially impossible to study them using X-ray crystallography. Although NMR can be a daunting technique for those outside of the field, it is extremely powerful, and arguably the only technique in the biochemist's toolbox to determine both global and per residue structural properties of an IDP without resorting to mutagenesis. A benefit of NMR compared to crystallography is that it is not an “all or nothing” technique; the researcher can decide how much NMR data collection is required to answer a particular question. Determining, for example, whether the protein binds a ligand and with what affinity, theoretically requires only one NMR experiment (Mittermaier and Meneses, 2013), whereas determining the ensemble structures of an IDP would require multiple experiments (Marsh and Kay, 2012). Experimental questions between these two extremes include examples, such as measuring the dynamics to quantify the relative amounts of disorder, determining which specific residues are involved in ligand binding, and whether those residues are gaining structure in the presence of a ligand.

The assumption in this paper is that sequenced-based bioinformatic methods have already predicted that the protein of interest is likely to be disordered. Many different approaches and programs exist (Dosztányi et al., 2005; Obradovic et al., 2005; Prilusky et al., 2005); for a recent review on IDP predictors, see Li et al. (2015). As well, the researcher can search databases which contain sequences of disordered proteins (Sickmeier et al., 2007; Oates et al., 2013; Fukuchi et al., 2014; Potenza et al., 2015), or search the pE-DB, which contains structural ensembles of IDPs and the data used in their determination (Varadi et al., 2014).

**Figure 1:**
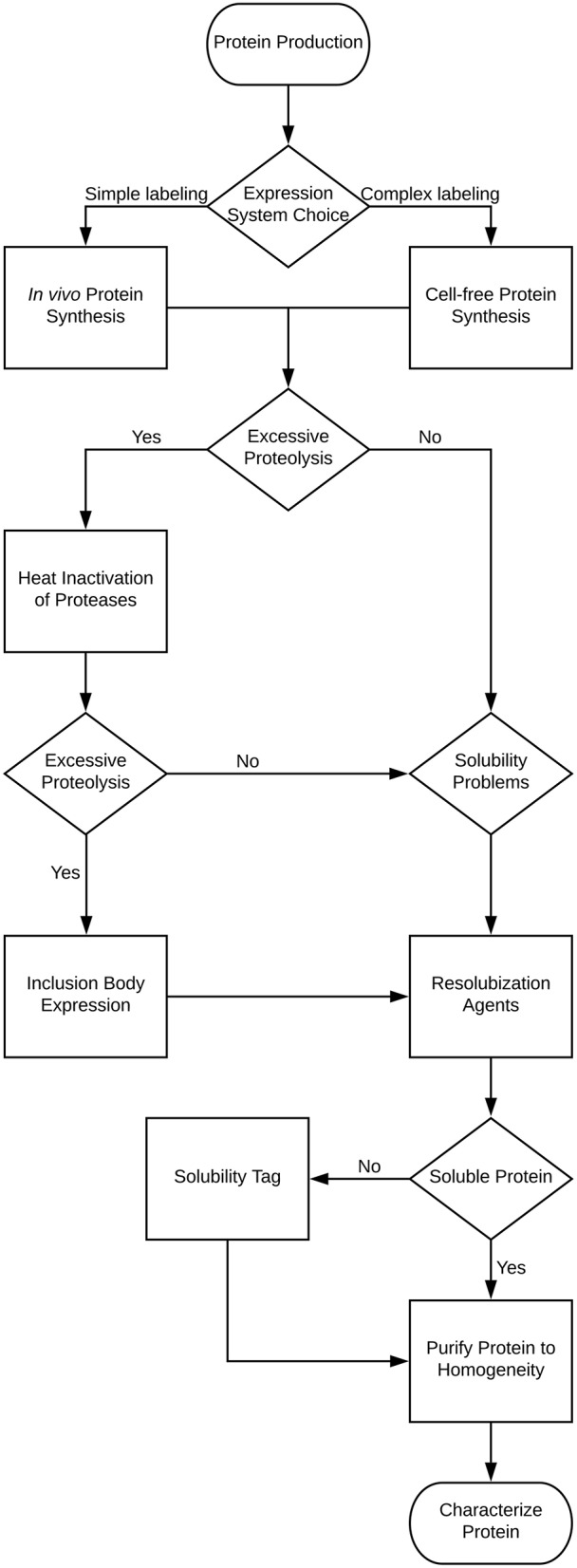
Decision tree for the expression and purification of an intrinsically disordered protein.

For NMR characterization, it is necessary to produce and purify the IDP from recombinant sources. While NMR has long been done on protein extracted from natural sources, for the most part studying IDPs will require protein labeled with stable isotopes, such as ^15^N and ^13^C. This guide is therefore written to cover the major steps with potential problems and decisions you may encounter in this process, with the problems and solutions being introduced at the point at which they would typically be discovered. Several of these methods are also applicable to ordered proteins as well, but where appropriate, specific mention is made of problems affecting disordered proteins. A decision tree of the overall process and the methods mentioned in this review is shown in Figure 1. Note that some methods are exclusionary to one another; Table 1 contains a process compatibility and applicability chart as guidance.

**Table 1:** Process compatibility and applicability chart.

**Cell-free expression**	**X**					
Solubility tag	√	√				
Heat inactivation of proteases	√	√	X			
Inclusion body directed expression	√	X	X	X		
Re-solubilization agents	√	X	X	X	√	
Insoluble tag removal	√	X	X	X	√	X
	Minimal media	Cell-free expression	Solubility tag	Heat inactivation of proteases	Inclusion body directed expression	Re-solubilization agents

*The chart lists which techniques are compatible or are applicable to the various techniques discussed in the text*.

## Gene Design and Recombinant Expression

### No cDNA Is Available for the IDP Gene

The first step for protein production in a recombinant host will be to obtain a cDNA encoding the disordered protein. This will, naturally, be the same for IDPs as for ordered proteins. The source DNA may be genomic, and need to be PCR amplified and manipulated using routine molecular biological approaches to incorporate it into a plasmid. One method, while not new but becoming increasingly affordable, is the “clone-by-phone” approach (Calçada et al., 2015), where the protein sequence is submitted to a commercial service, and for a fee a plasmid is sent in return. A major advantage of this approach is that the sequence can be optimized for recombinant host expression, which is not necessarily the same as the DNA source species. This point is especially relevant when cloning genes from eukaryotic organisms for expression in prokaryotic systems; codon usage can be very different, which has a dramatic effect on expression levels (Makrides, 1996). Although several commercial bacterial strains that contain a plasmid that encodes for rare codons are available, they do not include other benefits of a completely synthetic gene, such as optimizing mRNA secondary structure, removing potential RNase cleavage sites, optimizing ribosomal binding sites, improving transcription termination and increasing translational efficiency (Pfleger et al., 2006).

### Choosing the Expression System

The most popular system by far for recombinant protein expression is *E. coli*, due to its low cost and ease of use. Other host systems, such as yeast, insect, and plant cells, have become more viable as expression systems for NMR (Yanaka et al., 2018), but will not be discussed here. The specific *E. coli* strain choice will depend on its purpose (Makino et al., 2011). For protein expression, finding the optimal strain depends mainly on two points: the choice of induction system and codon usage. For the latter, various *E. coli* strains exist [e.g., Rosetta (DE3)] that contain a plasmid that encodes rare tRNAs. With respect to induction systems, the most popular system is the BL21(DE3) strain (Rosano and Ceccarelli, 2014), which uses lactose analogs (e.g., Isopropyl β-D-1-thiogalactopyranoside, IPTG) to induce expression. Other expression systems are available (Rosano and Ceccarelli, 2014), but in general do not give superior expression levels compared to BL21(DE3) and its derivatives. A researcher may wish to screen several different plasmids with different tags encoded in the plasmid to facilitate expression and purification. In this case, it is best to consider a high throughput system that uses ligase independent cloning methods (e.g., Gateway or TOPO) to simplify and accelerate the cloning process (Calçada et al., 2015).

For plasmid storage, it is highly recommended to use a strain that is unable to express the plasmid gene. Even in the absence of induction, leaky expression can cause host stress, and possibly introduce mutations into the plasmid that will affect the protein sequence or its expression levels.

### The Expression of an Isotopically Labeled Disordered Protein Results in a Low Yield

For advanced NMR techniques, there is the need for isotopic labeling, generally at minimum using a ^15^N source, such as ammonium chloride. This label is required to acquire an ^15^N-heteronuclear single quantum coherence (^15^N-HSQC) spectrum, which is often used as an initial experiment to see whether more complex and involved NMR experiments are feasible (see section Protein Characterization by NMR). Producing labeled proteins in a bacterial host typically means the use of minimal media, with M9 medium being the most common choice (Paliy and Gunasekera, 2006). The challenge with NMR is that it is a rather insensitive spectroscopic technique, often requiring milligram-scale quantities of proteins, and therefore large volumes of labeled media. Many different approaches to producing optimal amounts of protein in minimal media have been discussed; a particularly effective and simple method has been proposed by Marley et al. (2001). In this protocol, the cells are grown in a rich medium (for example, LB or 2xYT) until a relatively high cell density has been achieved. The cells are then removed from the rich media by centrifugation and transferred to the labeled media. After waiting for one hour to allow unlabeled proteins and metabolites to be cleared, expression can be induced. This method combines the advantages of growing in rich media to obtain a high density of cells with the cost-efficient use of labeled media for the actual protein synthesis.

### After Initial Expression Optimization, the Protein Production Is Still Low

For proteins that are difficult to express in minimal media, a commercially sourced, rich, labeled media can be used to obtain good bacterial growth (Verardi et al., 2012). However, this option is used infrequently due to its very high cost. An alternate method combines the advantages of rich media with the lower cost of minimal media (Rupasinghe et al., 2007). As shown in a technical report (Rhima et al., 2013), the supplementation of M9 media with some rich, labeled media led to faster growth, higher cell density and higher expression levels. Positive effects are observed even with 1% supplementation, with 5–10% leading to greater and maximal effects.

In most cases, unlabeled rich media can be used to test the effect of M9 media supplementation before committing to labeled rich media. If using small scale cultures to test expression yields, it is recommended to use 50 mL of medium in a 250 mL shaking flask. In our experience, 5 mL of culture in a test tube does not accurately mimic the aeration and growth conditions of a larger (≥500 mL) media volume.

### The IDP Is Toxic to the Cells

Sometimes, the expression of recombinant protein can be detrimental to cell growth, in essence they are considered to be toxic. Two strains that can help overcome expression problems are the C41(DE3) and C43(DE3) strains (Miroux and Walker, 1996). These *E. coli* BL21(DE3) derivatives can overcome issues with transformation and expression toxicity, where in some cases the severe overproduction of mRNA causes ribosomes to be highly occupied, and thus cause translation to stall. For problems with transformation, the C43(DE3) was shown to have higher plasmid stability for protein genes that were problematic in BL21(DE3) (Dumon-Seignovert et al., 2004), while for ribosome stalling, both C41(DE3) and C43(DE3) have been shown to reduce mRNA levels several fold (Miroux and Walker, 1996).

Alternatively, a cell-free expression system can be used (Hoffmann et al., 2018). The significant advantages of this system over in cell expression include an ability to deal with protein toxicity, preventing scrambling of isotopically labeled amino acids, and a capability to introduce post-translational modifications. Several different systems can be used, but the most popular two are *E. coli* and wheat germ lysates (Hoffmann et al., 2018). With respect to IDPs, a cell-free system offers advantages in that it can reduce damage by proteolysis (see section The Expressed Protein is Cleaved), and the use of specific amino acids labeling can help with the lack of dispersion problem (see section Protein Characterization by NMR). The latter was specifically used in the expression of the Neh2 domain, an intrinsically disordered protein which suffered from severe overlap (Tong et al., 2008). In that particular case, the researchers were looking to specifically label glutamine and glutamate residues with ^15^N, without the amino acids being metabolically scrambled to other amino acids by transamination reactions.

A survey of the expression of 3,066 human proteins found that IDPs were generally good candidates for cell-free synthesis (Kurotani et al., 2010). The work suggested that the highly soluble nature of IDPs results in expression success. It is possible, however, that self-aggregation prone IDPs (section The IDP is Insoluble) may not fare well with this approach. This result is somewhat contradicted by another survey of IDP production in cell-free synthesis (Tokmakov et al., 2015), where they found that the soluble nature of IDPs meant an increase in expression success, but resulted in less total detectable expression, possibly because the disordered proteins are being targetter for proteolytic degradation. Using IDPs in a cell-free expression system is possible, but likely best fits for cases where residue specific labeling or specific post-translational modifications are required.

### The Tag Interferes With the Function of the IDP

The presence of an added tag may interfere with the structure and/or function of the IDP in a subtle way that cannot be easily detected until after extensive data collection and analysis. It is therefore advisable to design the gene from the beginning so that the tag can be cleaved during the purification process, even before there is any evidence of a problem. Fortunately, most tags encoded in commercial plasmids also encode a proteolytic cleavage site. While helpful, in most cases extra residues will still remain after treatment, where the exact sequence varies between the different proteases (Terpe, 2003).

Three common tags that are used to help with protein expression include maltose-binding protein (Kapust and Waugh, 1999), glutathione-S-transferase (Smith and Johnson, 1988), and thioredoxin A (TrxA) (LaVallie et al., 2000). TrxA has been successfully used in aiding disulfide bond formation (Lebendiker and Danieli, 2014), though this is unlikely to be an issue for IDPs given the scarcity of cysteine residues in their sequences. It has also been shown to rarely contribute to solubility (Lebendiker and Danieli, 2014), and may promote aggregations through its propensity to dimerize (see section The IDP is Insoluble). This effect was seen in a study with a plant antivirulence protein, where the thioredoxin-fused disordered protein gained solubility only after the gene of interest was altered (Schneider et al., 2010). These results all suggest that care must be taken when using the TrxA tag with an IDP.

An alternative tag system that we found to help with expression of IDPs is the SUMO-tag (Marblestone et al., 2006). In this case, the tag is an entire SUMO domain that also includes an N-terminal His-tag. The two advantages of this tag are that the cleavage is carried out by a highly specific SUMO-protease, which recognizes the entire SUMO domain, rather than just a short recognition sequence, and the other advantage is that the protocol leaves a “native” (as in user-defined) N-terminus on the IDP. While commercial sources for the SUMO protease are available, we have found it cost efficient to produce our own (Reverter and Lima, 2009; Patel and Graether, 2010).

## IDP Purification

### The Expressed Protein Is Cleaved

Given the disordered nature of IDPs, it is not surprising that they are often excellent substrates for proteases in the recombinant host. Using protease inhibitors and handling samples at low temperatures does reduce the amount of cleavage, but the high proteolytic sensitivity of IDPs often requires additional care; in fact, cleavage has even been observed inside the cell (Tolkatchev et al., 2010). Exporting to the media, where there is a lack of proteases, is a possible solution. The challenge there is that one must employ a strong and efficient capture step that is capable of handling large volumes (Linn, 2009), and in some cases cleavage was still found to occur (Goda et al., 2015). Two other options that are applicable to IDPs are described in the following sub-sections.

#### Option 1—Heat Inactivation of Proteases

One common method to deal with proteolytic cleavage is to boil the bacterial lysate as a first step after rupturing the cells. Heating can be used because fully disordered proteins have no structure to lose. An additional advantage is that the heating causes aggregation of many cellular proteins, which can be simply removed by centrifugation. To improve the process, rapid cooling can be performed with a salt water bath to promote aggregation (Kalthoff, 2003). In contrast, most IDPs stay soluble because of their high number of charged residues and fewer hydrophobic ones (Kalthoff, 2003).

The problem with boiling lysates is that proteolysis can still occur during the mechanical or chemical lysis step. A solution has been to combine cell lysis and boiling into one (Kalthoff, 2003; Livernois et al., 2009; KrishnaKumar and Gupta, 2017). Proteolytic damage is significantly reduced and, in some cases, the resulting sample can be nearly as pure as a His-tagged purified protein, with the added advantage of not needing to subsequently remove the tag (Livernois et al., 2009). Aggregates can be removed through a combination of ultracentrifugation, followed by sample filtration with a 0.2–0.8 μm syringe filter. I recommend filters designed specifically for samples with high-solids content, such as the Whatman GD/X system, to prevent the need for multiple filters in one preparation.

One downside to heat inactivation of proteases is that boiling the IDP increases the chance of a Maillard modification occurring (Kalthoff, 2003). To eliminate this possibility, the molecular weight of the purified protein can be measured. Note that the N-terminal Met is often cleaved from a bacterial recombinant protein (Makrides, 1996). Lastly, some IDPs may not be completely disordered, in which case the heat treatment could disrupt their structure. It is highly recommended in those cases to check that the protein is still native through a functional assay or by assessing its structure, such as by circular dichroism (CD), to compare samples that have and have not been heat treated (Kalthoff, 2003; KrishnaKumar and Gupta, 2017).

#### Option 2—Directed Expression Into Inclusion Bodies

Several research groups have purposefully directed the expressed IDP into inclusion bodies, where active proteases are not found, and any contaminating cellular proteases picked up during lysis cannot function on the recombinant protein because it is in the insoluble state. Generally, inclusion bodies are avoided for ordered proteins, since it is often a major challenge to refold them (Singh and Panda, 2005). With fully a disordered protein, this is obviously not a concern. The targeting of IDPs to inclusion bodies is performed through the use of a fusion construct (Hwang et al., 2014). Removing the tag, however, is not necessarily a trivial problem, and is discussed in section The Tag Needs to be Removed From an Insoluble IDP.

### The IDP Is Insoluble

In some cases, IDPs can end up in an inclusion body, even in the absence of a specific tag (Churion and Bondos, 2012). While it may seem counter-intuitive for a highly polar and charged protein to be insoluble, it has been suggested that the propensity for IDPs to be involved in protein-protein interactions may promote this behavior. The ability of IDPs to readily form hydrogen bonds, many charged residues that can contribute to electrostatic interactions, and entropic factors can contribute to IDP aggregation (Linding et al., 2004). In some cases, the IDP may become soluble using resolubilization agents, and/or after contaminating proteins have been removed. SDS-PAGE of soluble and pellet fractions of crude lysates provides an effective way to quickly scan resolubilization conditions through the addition of different classes of resolubilization agents (Churion and Bondos, 2012). Broadly, the classes can be divided into salts (e.g., NaCl), stabilizers (e.g., glycerol), mild chaotropes (e.g., low concentrations of urea), amino acids (e.g., arginine), and detergents (e.g., Tween-20). Note that the concentration of the agent may also need to be screened. It is advisable to not use denaturants stronger than necessary, not because of concern for problems with protein refolding, but to prevent protein modification. Guanidinium hydrochloride is ideal since it causes minimal modification of proteins and is compatible with many metal-affinity purification methods (Hwang et al., 2014). The downside is that it is not readily compatible with SDS-PAGE. Urea is compatible with gels, but there is a danger of covalently modifying the IDP by carbamylation of the amino groups (Hwang et al., 2014).

Another way to potentially improve solubility is to express the IDP as a fusion with a highly soluble protein as a tag (see section The Tag Interferes With the Function of the IDP).

### The Tag Needs to be Removed From an Insoluble IDP

For IDPs targeted to inclusion bodies, the tags need to be removed to resolubilize the protein. The previous advantage of proteases being inactive in inclusion bodies and in the presence of resolubilization agents (i.e., denaturants) now becomes a disadvantage. One solution has been to use chemical cleavage, which is not affected by the presence of denaturants. The best known reagent is cyanogen bromide (CNBr), which will efficiently cleave after Met as long as it is not followed by Ser or Thr residues, though methods are available to reduce the effect of this problem (Kaiser and Metzka, 1999). For cases where Met residue(s) are located internally in the IDP sequence, other approaches have been developed. One promising new method cleaves the sequence SRHW by nickel ion catalysis (Zahran et al., 2015). The conditions are alkaline (pH 9.0) and the cleavage is performed at an elevated temperature (45°C), neither of which are an issue for disordered proteins. One concern is that cleavage occurs N-terminal to this sequence, resulting in the N-terminus of the IDP containing these four extra residues, and hence potentially affect its structure or function.

An alternate approach involves the use of an autoprotease (Goda et al., 2015). In this method, the N^Pro^ fusion sequence (EDDIE), which also contains an autoprotease from the classical swine fever virus, is tagged to the IDP. During refolding (i.e., during removal of the denaturant), the autoprotease becomes active again, and cuts such that the recovered IDP has a native N-terminus. The researchers tested 10 different IDPs and found that all of them worked, regardless of the organism from which they were originally derived, suggesting that their approach should work with many different disordered proteins (Goda et al., 2015).

### The Protein Needs to be Further Purified

While methods, such as those listed above (direction to inclusion bodies, heat inactivation of proteases) can result in very pure protein samples, in most cases, additional separation steps will be necessary. For IDPs, this can in large part be similar to that for ordered proteins, but the unusual sequence composition of IDPs allows for different considerations to be made in selecting optimal chromatographic methods.

The use of His-tags has already been mentioned previously, since this tag is often present in purification tags (sections The Tag Interferes With the Function of the IDP). Of additional note is that some IDPs are naturally rich in His residues, a property that has been exploited in the purification of disordered plant stress proteins known as dehydrins (Graether and Boddington, 2014). In this example, while the traditional/engineered hexa-His sequence was not present, the clustering of pairs of His residues was sufficient to allow for purification by a nickel-affinity column (Hernandez-Sanchez et al., 2014). Nevertheless, it is not enough to result in near homogeneity, and additional purification steps are often necessary.

Other typical chromatographic resins used in protein purification include ion-exchange (IEX) and size-exclusion chromatography (SEC), and again the unusual sequence composition of IDPs can often be exploited. With respect to SEC, the most interesting IDP property is that their lack of a hydrophobic core results in them having a large hydrodynamic radius compared to globular proteins of the same length (Uversky, 2002b). Therefore, IDPs will migrate through a SEC column much faster, possibly resulting in better separation from the contaminants. For IEX, it should be noted that it is often a useful technique for IDPs because they often have a large net pI (either acidic or basic) compared to ordered proteins (Uversky et al., 2000). Therefore, IEX on IDPs can be performed using more stringent binding conditions (higher salt) to prevent non-specific binding of contaminant proteins, and they will generally elute at higher salt concentrations.

### Protein Purity and Concentration Determination

In most cases, protein purity will be assessed during the purification process by protein gel electrophoresis. While a simple and common technique, it relies on protein separation based on size; this is an issue for IDPs, where their hydrodynamic radii are typically larger than that of a globular protein of similar length even in the presence of a denaturant, such as SDS. Another approach that we have used to analyze disordered protein purity is by analytical HPLC. In this protocol, a small (100 μg scale) amount of material is loaded on a reversed-phase C18 column. Absorption should be monitored at 214 nm, since many IDPs are low in or contain no aromatic amino acids that absorb near 280 nm. Using HPLC has the advantages over gel electrophoresis of detecting small molecule and peptide contaminants that would otherwise run off a gel and/or may be inefficiently stained by the dye. Peak integration can then be used to quantify the percent purity.

IDP concentrations are often a challenge to quantify by standard biochemical techniques (Szollosi et al., 2007). The gold standards for protein concentration determination are amino acid analysis and Kjeldahl analysis, but these techniques are not optimal for routine use in most labs. A recent analysis compared several different methods for determining the concentration of ordered and disordered proteins (Contreras-Martos et al., 2018). The researchers found that while the concentration of the ordered proteins using the Bradford and BCA assays were usually within 30% of the expected value, the disordered proteins show typically a >60% difference, with extreme cases having >80% difference from the expected amount. Their key result showed that the ninhydrin assay method is the best choice for determining the concentration of an IDP (Contreras-Martos et al., 2018).

## Protein Characterization by NMR

### ^15^N-HSQC—An Initial NMR Experiment

After the protein has been successfully purified, its structural characterization can begin. Most NMR assignment experiments used with IDPs are the same as those used for ordered proteins. The reader is referred to introductory information on using NMR to assign atoms of a protein (Teng, 2013). In this section, I focus on methods that are used as an initial experiment to assess the feasibility of running more complex and involved NMR methods on the IDP.

With ordered proteins, the ^15^N-HSQC experiment is often the first experiment run in order to compare the number of observed residues vs. the expected. The ^15^N-HSQC is easy and relatively quick to collect (typically on the order of minutes), simple to interpret (mainly counting the number of observed peaks) and requires only the relatively inexpensive ^15^N label. The result, the ^15^N-HSQC “fingerprint” of a protein, gives an idea of the overall quality of the sample (Brutscher et al., 2015), and also allows for a rapid scan of multiple conditions (pH, salt, temperature, ligands etc.) before starting longer and more complex NMR experiments. While useful as an initial scan for IDPs, there can be a number of issues as outlined below.

### The ^15^N-HSQC Spectrum Is Highly Overlapped and/or Many Residues Are Missing

Unfortunately the ideality of the ^15^N-HSQC experiment is compromised in several ways when studying disordered proteins; the most significant of which is the lack of dispersion (i.e., data spread). Most residues in an IDP are exposed to the same solvent environment, resulting in many of the peaks being partially or even mostly overlapped (Nováček et al., 2013). Additional complications include the fact that IDPs are often rich in Pro, which lack an amide ^1^H, and hence would give no signal in the ^15^N-HSQC spectrum, and that the low sequence complexity of IDPs can add to the severe signal overlap problem.

An alternative, early stage experiment that overcomes many of the limitations of the standard ^15^N-HSQC experiment are the “CON” experiments (Goradia et al., 2015; Gibbs and Kriwacki, 2018). This series of NMR experiments correlates signals from ^13^C atoms with ^15^N atoms. The most significant difference is the use of direct ^13^C detection instead of ^1^H, which provides several advantages: there is no concern about proton exchange with the solvent (which is especially prevalent in IDPs and causes signals to be weak or disappear); line broadening of the ^1^H signal, caused by conformational exchange (changes in structure, despite the disorder); and Pro residues are observed (Brutscher et al., 2015; Goradia et al., 2015). The disadvantage of these experiments is that they require the protein to be labeled with both ^15^N and ^13^C.

### The NMR Structure of the IDP Needs to be Determined

After assigning as many atoms as possible, an initial examination of the structure of the IDP, at least in terms of secondary structure and on a per residue basis, can be made with a detailed analysis of the chemical shifts. Because of the disordered nature of IDPs, their chemical shifts will be very close to coil values (Kashtanov et al., 2012), but differ slightly because even a disordered protein will transiently occupy some states more frequently than others. Two programs that can be used to analyze the chemical shifts are the secondary structure propensity (SSP) (Marsh et al., 2006) and δ2Δ (Camilloni et al., 2012) programs. They combine the secondary chemical shifts into a fractional measure of secondary structure (coil, α-helix, β-sheet). The main difference between the two programs is that δ2Δ also includes polyproline type II helix secondary structure.

A thorough interpretation of the “structure” beyond secondary structure propensity of an IDP is an involved process. Generally, there are several approaches (Showalter, 2014), but in all cases it must be understood that the resulting structures are just possible conformers of the protein, rather than specific structural snapshots. The method works by first generating a very large number of chemically plausible structures, and then selecting a subset of that population based on structural data as representative conformers to get a sense of what the IDP may look like. The more NMR restraints collected, the greater the selection constraints, and, therefore, the more likely the generated structures are a good representation of reality. A list of the different types of NMR experimental constraints that can be collected for structural analysis of an IDP are listed in Marsh and Kay (2012).

## Author Contributions

The author confirms being the sole contributor of this work and has approved it for publication.

### Conflict of Interest Statement

The author declares that the research was conducted in the absence of any commercial or financial relationships that could be construed as a potential conflict of interest.
